# Analysis of Three-Phase Structure of Epoxy Resin/CNT/Graphene by Molecular Simulation

**DOI:** 10.3390/polym12081821

**Published:** 2020-08-13

**Authors:** Shun Naito, Jun Koyanagi, Takuji Komukai, Toshikazu Uno

**Affiliations:** 1 Department of Materials Science and Technology, Graduate school of Tokyo University of Science, Tokyo 125-8585, Japan; 8220535@ed.tus.ac.jp; 2 Nitta Corporation, Yamtokohriyama-Shi, Nara 639-1085, Japan; Ta_Komukai@nitta.co.jp; 3 Yonex CO., LTD., Nagaoka-City, Niigata 949-5123, Japan; t-uno@yonex.co.jp

**Keywords:** molecular dynamics, three-phase structure, CNT, graphene

## Abstract

In this study, the three-phase structure consisting of epoxy resin, carbon nanotubes (CNTs), and graphene, which is assumed to be the surface of carbon fiber, was simulated using molecular dynamics. Models in which the CNT number and initial position of CNT are varied were prepared in this study. Relaxation calculation for each three-phase model was implemented, and the movement of molecules was investigated. When CNTs are located between the graphene and epoxy at initial, how the epoxy approaches to graphene was discussed. Besides, interaction energies between CNT/graphene, CNT/epoxy, and graphene/epoxy were evaluated after relaxations. The value of the interaction energy between two individual molecules (epoxy resin and graphene, CNTs and graphene, epoxy resin and CNTs) among three-phase structure were obtained, respectively, and those mechanisms were discussed in this study.

## 1. Introduction

Carbon fiber reinforced plastic (CFRP) uses resin as a base material and carbon fiber as a reinforcing agent. Lightweight, high strength, and high stiffness are the essential properties of CFRP. Therefore, it is used in aircraft, wind turbines, satellites [1]. The excellent performance accruing from these properties is hugely reliant on the interfacial adhesion between the carbon fiber and resin [2,3]. Presently, composite materials to which CNTs are added to CFRP are attracting attention as the addition of CNTs improves mechanical properties. Specifically, CNTs dispersion improves various properties, including tensile properties and fracture toughness. These materials are used in aerospace, transportation, wind turbine blades, electromagnetic interference shielding, and expensive sporting goods, etc. [4,5,6,7,8,9,10,11,12,13,14,15,16,17,18,19]. It is very important to understand whether CNT is attracted more by carbon fiber or epoxy resin in order to control the CNT dispersion. Molecular simulation is one of the methods to discuss such nanoscale behavior.

Recently, many studies have been conducted using molecular dynamics (MD) simulations, including several studies on the molecular interface that is related to a component of materials [20,21,22,23,24,25,26,27,28,29,30,31,32,33,34,35,36,37,38,39]. Jin Y et al. [36] investigated the interfacial interaction between functionalized graphene sheet (FGS) and polyethylene (PE) using the reactive force field (ReaxFF) and showed that several interfacial chains were attached to the FGS and extracted from the polymer matrix. The strength of the interaction is influenced by the size of the functional group, and the formation of covalent and hydrogen bonds through the interface makes the interaction more powerful. Arash B et al. [37] observed that the interaction between CNTs and a polymer matrix greatly affected the material properties. The results of this study showed that Young’s modulus of polymethylmethacrylate (PMMA) polymer matrix composites reinforced with CNTs increased significantly, up to 16 times the stiffness of pure PMMA polymer materials. Li Y et al. [38] created a model with CNTs and polymer matrix and a model with graphene and polymer matrix, and the interfacial interaction was obtained via consideration of interfacial friction. Polymer composites reinforced by the graphene sheet have larger interfacial interactions between molecules than those reinforced by CNTs. Polymer composites reinforced by graphene sheet have an 18% higher Young’s modulus, 8.7% higher tensile strength, and 5% higher interfacial energy than polymer composites reinforced by CNTs. The enhancement of mechanical properties is explained based on the interfacial interaction energy and total van der Waals energy. From investigating molecular interaction in a two-phase structure, it has been found that the state of the molecular interface affects the material properties. 

Yu B et al. [39] emphasized the π–π interaction between CNTs and polymers. Regarding interfacial adhesion between aromatic polymers and single-walled CNTs, interfacial interactions with polymers that have benzene in the main chain are stronger than those with benzene as side groups. The polymer chain spreads along the surface of the CNTs and, thus, forms a larger area of π–π stacking, which improves interfacial interaction. A study on π–π interaction shows that there are three types of interactions between benzene: edge-to-face T-shape, parallel displaced, and cofacial parallel stacking of the π–π interactions between benzene systems. The parallel displaced and T-shaped are the most stable, while the cofacial parallel stacked is undesirable because the interaction is stronger due to the angle being smaller and closer to parallel than for benzene with larger angles [40,41]. It has been shown that it is necessary to understand the π–π interaction in order to further clarify the composite material properties.

There are some studies on the interface of the graphene/CNTs/epoxy resin model [42,43]. Zhang Y et al. [42] reported the study with an emphasis on load transmission. The radius of the CNT and the distance between the CNT and graphene have little effect on the mechanical properties of composites. However, the position of the CNT has a specific effect on the shape of the area damaged during a tensile process. Sun S et al. [43] determined the interaction energy of the graphene interface in CFRP filled with CNTs using MD simulations. It was established that the addition of CNTs strengthened the interfacial adhesion between graphene and the polymer matrix. They reported the CNT radius, the distance between CNT and graphene, and multilayer CNTs had an effect on the interfacial adhesion. To determine the interaction in CNTs-enhanced CFRP, it is necessary to create a three-phase structure. However, not much research using MD simulation on the three-phase structure of graphene/CNTs/epoxy resin has been done so far. Furthermore, there are no studies on the interaction between two individual molecules in a three-phase structure. 

In this study, we focused on the interaction between two individual molecules in a three-phase structure and calculated the interaction energy between two individual molecules, considering the influence of another molecule on the three-phase structure. We emphasized the interaction between epoxy resin and CNTs, which are arranged in parallel with graphene, and also emphasized the π–π interaction between benzenes. We observed the behavior of CNTs and epoxy resin and compared the interaction energy between CNTs-epoxy resin and CNTs-graphene. Our overarching aim was to clarify the structure of graphene/CNTs/epoxy resin.

## 2. Simulation Method and Molecular Behavior

### 2.1. Modeling Each Molecule and Creating a Three-Phase Structure

We created a full atomic model. To model the three-phase structure, we prepared each molecule: epoxy resin (number of particles: 8960); three graphene sheets of cell size: (*x*, *y*, *z*) = (44.32, 38.38, 6.707) (Å); CNTs (4,4) and (8,8), with diameters of 0.5420 and 1.084 nm, respectively. J-OCTA 5.0 software (JSOL Corporation, Tokyo, Japan) was used for analysis and VSOP (JSOL Corporation, Tokyo, Japan) as the MD engine. J-OCTA5.0 enabled us to create CNTs and to input force field parameters easier. The DREIDING force field parameters were used [44], and the condition of the electric charge was neutral.

**Figure 1:**
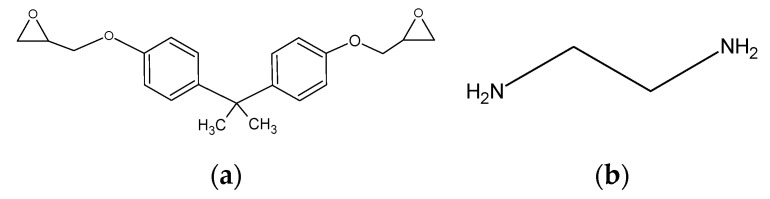
Molecular structure of bisphenol A diglycidyl ether (DGEBA) (**a**) and ethylenediamine (**b**).

**Figure 2:**
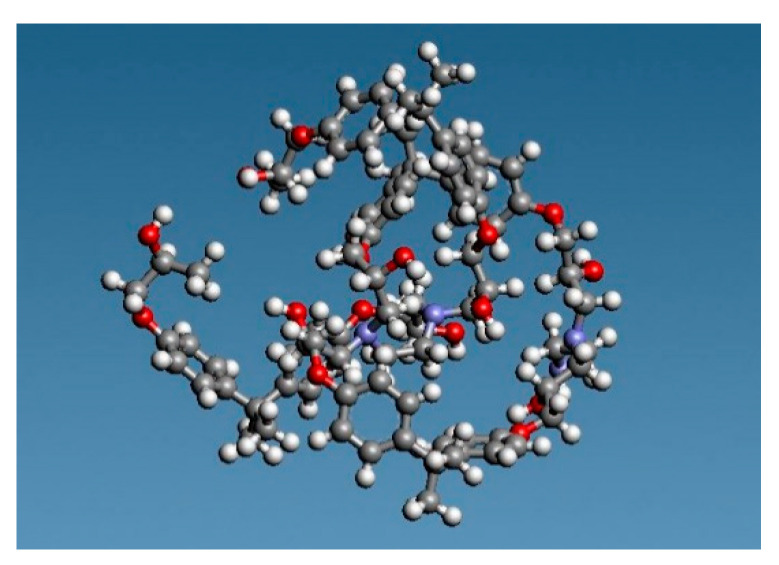
Molecular structure of an epoxy resin.

**Figure 3:**
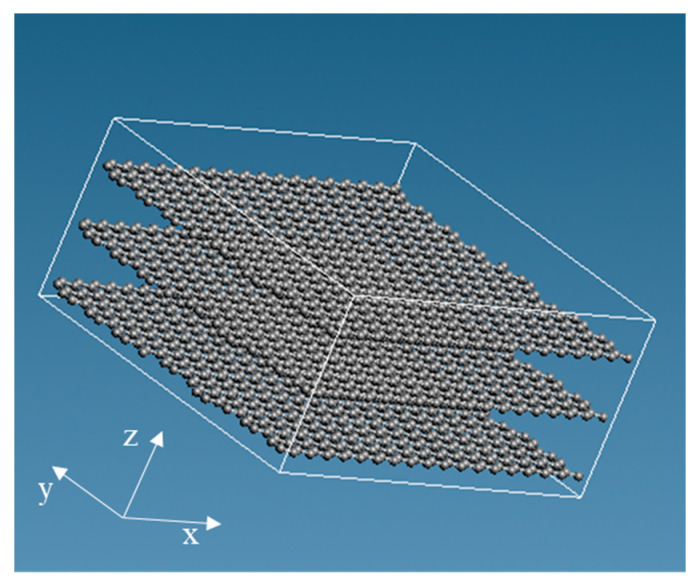
Molecular structure of graphene.

**Figure 4:**
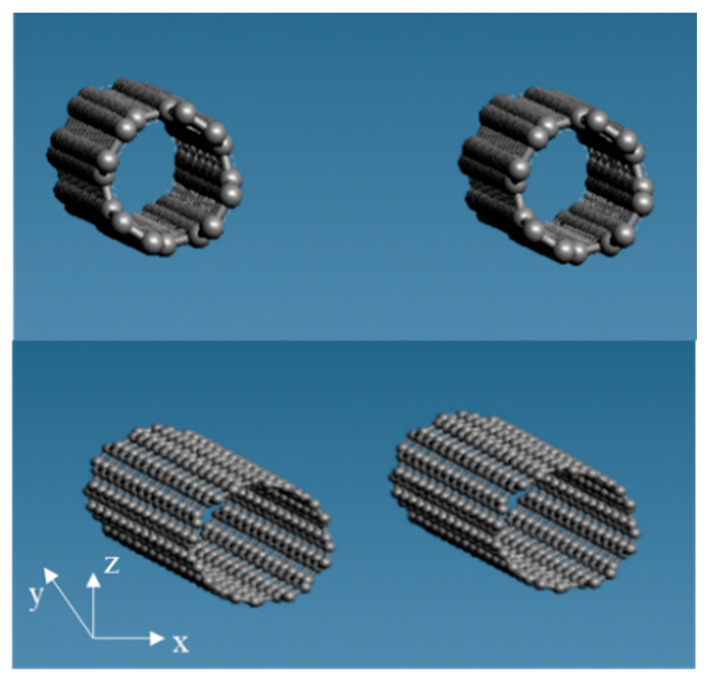
Molecular structure of carbon nanotubes (CNTs) (4,4) (top) and CNTs (8,8) (bottom).

We made epoxy resin using bisphenol A diglycidyl ether of bisphenol A (DGEBA), with a molecular weight of 344.4, as an epoxy monomer, and ethylenediamine (EDA), with a molecular weight of 60.10, as the curing agent. The structural formula of each reagent is presented in Figure 1. Two DGEBA, one EDA, two DGEBA, and one EDA were bonded in this order to produce an epoxy resin. After that, the initial structure was relaxed, and 40 of those were randomly arranged to produce epoxy resin with 8960 particles. The molecular structure is shown in Figure 2. The red, blue, gray, and white spheres in Figure 2 represent oxygen, nitrogen, carbon, and hydrogen atoms, respectively. We created the graphene structure. Each graphene sheet had a size of (*x*, *y*) = (44.32, 38.38) (Å). We created the graphene with 1944 carbon atoms. Subsequently, three graphene sheets were stacked in the *z*-direction with cell size: (*x*, *y*, *z*) = (44.32, 38.38, 6.707) (Å). The molecular structure is shown in Figure 3. Finally, when the three-phase structure was created, stacking three graphene sheets could reduce the interaction between CNTs above the graphene and epoxy resin below the graphene. We made CNTs, creating two CNT molecules with chiral indices (4,4) and (8,8) with diameters of 0.5420 and 1.084 nm, to investigate the effect of different CNT diameter sizes on the interaction. We considered phasing after creating the structure, and the axial length was set to 3.838 nm to match the length of the graphene sheet in the y-axis direction. The initial structure was relaxed, as shown in Figure 4. The gray spheres in Figure 3 and Figure 4 represent carbon atoms.

**Figure 5:**
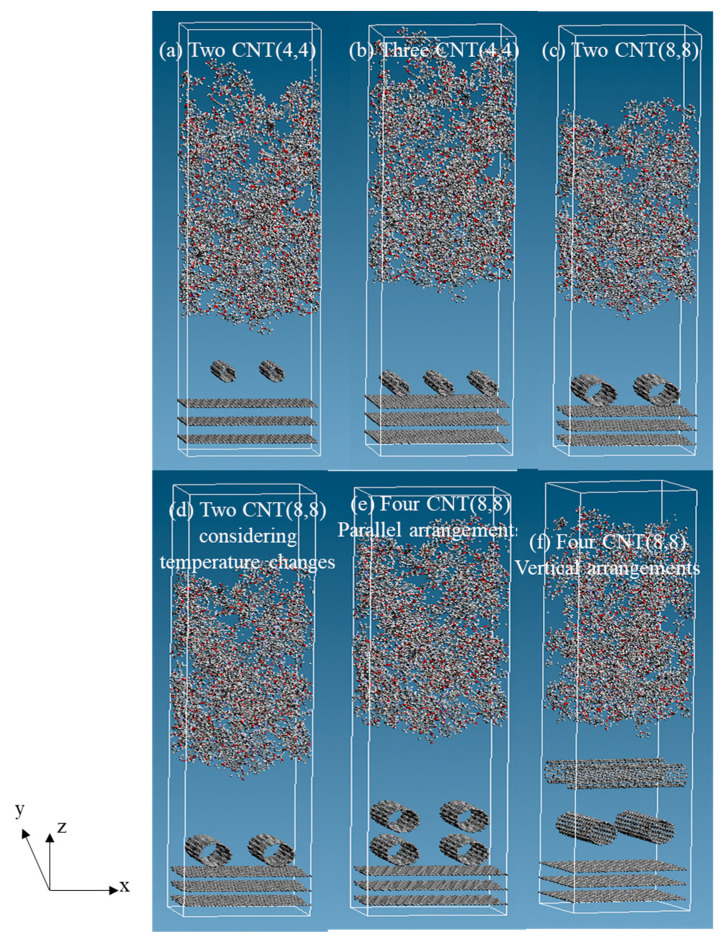
The six three-phase molecular structures before relaxation (considering periodic boundary conditions in the *x*, *y*, and *z* directions). (**a**) two CNTs (4,4); (**b**) three CNTs (4,4); (**c**) two CNTs (8,8); (**d**) two CNTs (8,8), considering temperature changes; (**e**) four CNTs (8,8) parallel arrangements; (**f**) four CNTs (8,8) vertical arrangements.

We arranged the epoxy resin, CNTs, and graphene from top to bottom in the *z-*direction and created a total of six types of three-phase structures: (a) two CNTs (4,4); (b) three CNTs (4,4); (c) two CNTs (8,8); (d) two CNTs (8,8), considering temperature changes; (e) four CNTs (8,8) parallel arrangements; (f) four CNTs (8,8) vertical arrangements. In the actual structure, CNTs adhered to the surface of the carbon fiber in a dispersed state. Models with CNTs (8,8) parallel and vertical arrangements were prepared, respectively, to simulate a similar structure. The molecular structures are shown in Figure 5. For the model with CNT (4,4) in Figure 5a,b, the cell size was (*x*, *y*, *z*) = (44.32, 38.38, 160.0) (Å). For the model with CNT (8,8) in Figure 5c–f, the size in the *z*-direction was 190.0 Å. CNT (8,8) had a larger diameter than CNT (4,4). Thus, we made room for the entire structure by extending the *z-*direction of the cell. The graphene placed at the bottom was fixed. We considered periodic boundary conditions in the *x*, *y*, and *z* directions. Table 1 shows the dimensions of the simulation cell and a CNT diameter, the number of CNTs, how to arrange CNTs, temperature change values of each model.

### 2.2. Relaxation of the Three-Phase Structure

The relaxation calculation procedure for each three-phase structure is presented below:

(1) The canonical ensemble which is performed with constant volume, temperature, and number of particles (NVT ensemble) was created at a temperature of 300 K. Step time was 0.5 fs. The total number of steps was 1000. Relaxation time was 2 ns.

(2) The isothermal-isobaric ensemble (NPT ensemble) was set at a temperature of 300 K and an atmospheric pressure of 0.1 MPa in the *z-*direction. The step time was 0.5 fs. The total number of steps was 1000. Relaxation time was 2 ns. There was an exception; the initial temperature was set to 600 K for one model with two CNTs (8,8). Compression was performed, while the temperature was lowered from 600 K to 300 K to investigate the behavior and interaction energy difference when a temperature change from high to low occurred at constant pressure.

(3) The NVT ensemble was created at a temperature of 300 K. Step time was 0.5 fs. The total number of steps was 1000. Relaxation time was 2 ns. The model with four CNTs (8,8) arranged vertically was also examined with the relaxation time increased to 10 ns.

The procedure used the Nose-Hoover method for temperature control and the Parrinello-Rahman method for pressure control.

### 2.3. Molecular Analysis

After relaxation, the behavior of each molecule was observed, and the interaction energy was derived. The interaction energy between one molecule and other molecules was calculated using Equations (1)–(3), respectively.

(1) $$E_{G-\left(C/E\right)}=E_{\mathrm{Total}}-\left(E_{G}+E_{C/E}\right)$$

(2) $$E_{E-\left(C/G\right)}=E_{\mathrm{Total}}-\left(E_{E}+E_{C/G}\right)$$

(3) $$E_{C-\left(E/G\right)}=E_{\mathrm{Total}}-\left(E_{C}+E_{E/G}\right)$$

$E_{G-\left(C/E\right)}$ is the interaction energy between graphene and other molecules, which are CNTs and epoxy resin, $E_{E-\left(C/G\right)}$ is the interaction energy between the epoxy resin and other molecules, which are CNTs and graphene, $E_{C-\left(E/G\right)}$ is the interaction energy between CNTs and other molecules, which are epoxy resin and graphene, $E_{\mathrm{Total}}$, $E_{G}$, $E_{E}$, and $E_{C}$ represent the energy of the entire system, graphene, epoxy, and CNTs, respectively. $E_{C/E},\;E_{C/G}$, and $E_{E/G}$ represent the energy of CNTs and epoxy, CNTs and graphene, and epoxy and graphene, respectively. 

We also calculated the interaction energy between two individual molecules. The interaction energy between epoxy and graphene in the system was $E_{E-G}$. The interaction energy between CNTs and graphene was $E_{C-G}$. $E_{G-\left(C/E\right)}$ is the sum of $E_{E-G}$ and $E_{C-G}$ and is expressed as Equation (4). The interaction energy between epoxy and CNTs was $E_{E-C}$. $E_{E-\left(C/G\right)}$ and $E_{C-\left(E/G\right)}$ were expressed as Equations (5) and (6), respectively. We solved Equations (4)–(6) simultaneously. The interaction energy between two individual molecules was calculated using these three equations.

(4) $$E_{G-\left(C/E\right)}=E_{E-G}+E_{C-G}$$

(5) $$E_{E-\left(C/G\right)}=E_{E-G}+E_{E-C}$$

(6) $$E_{C-\left(E/G\right)}=E_{C-G}+E_{E-C}$$

After calculating the interaction energy values, we used absolute values for each of the interaction energy values to make it easier to compare them.

## 3. Results

### 3.1. Results of Molecular Behavior

**Figure 6:**
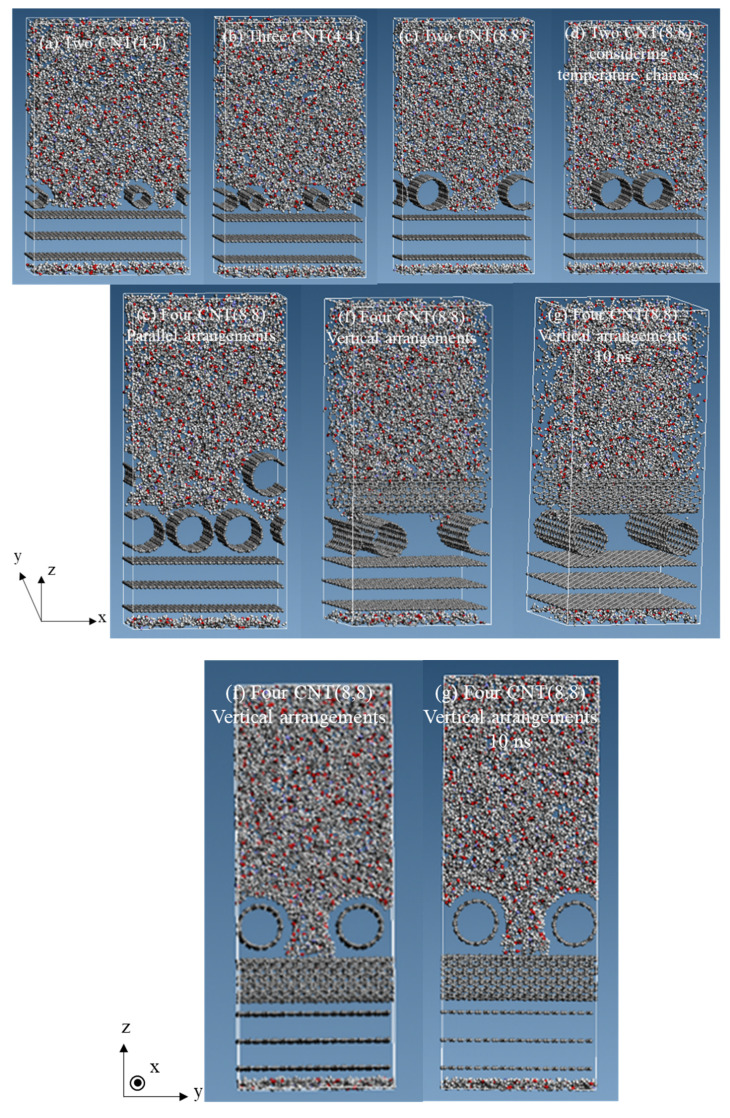
The six structure types after 2 ns relaxation and the four CNTs (8,8) vertical arrangements structure after 10 ns relaxation. (**a**) two CNTs (4,4); (**b**) three CNTs (4,4); (**c**) two CNTs (8,8); (**d**) two CNTs (8,8), considering temperature changes; (**e**) four CNTs (8,8) parallel arrangements; (**f**) four CNTs (8,8) vertical arrangements; (**g**) four CNTs (8,8) vertical arrangements, relaxation time is 10 ns.

We observed the molecular behavior during relaxation in the NVT ensemble. In all six structure types, immediately after starting relaxation, the epoxy resin above the CNTs was drawn toward the fixed graphene. Next, we confirmed the cell compression in the NPT ensemble. The structure after relaxation in the NVT ensemble is shown in Figure 6, respectively. We then observed the molecular behavior of each model and observed that epoxy resin penetrated in between the CNTs in the model with CNT (4,4) but did not penetrate in between the CNTs in the model with CNT (8,8) (Figure 6a–d). In Figure 6, it appears that epoxy resin penetrated between the CNTs. However, no epoxy resin penetrated between the CNTs because of considering the periodic boundary condition in the y-direction. In other words, epoxy resin penetrated in between CNTs with a small diameter, but not CNTs with a large diameter. This is because larger CNTs had more benzene rings, and the π–π interaction was stronger than in small-diameter CNT. Thus, CNTs with a large diameter did not move away from each other. Furthermore, there was no significant difference in molecular behavior based on temperature change. We also considered the models with four CNTs (8,8) parallel arrangements and four CNTs (8,8) vertical arrangements (Figure 6e,f). One CNT in the upper region penetrated the lower region in the model with four CNTs (8,8) parallel arrangements. Similarly, no epoxy resin penetrated in between the CNTs, and an epoxy resin covered one CNT at the top. For the vertically arranged CNTs (8,8), epoxy resin approached the upper CNTs region, but no epoxy resin reached the CNTs at the bottom of the structure. When the relaxation time was 10 ns, the epoxy resin did not go any further down (Figure 6g). From this result, it can be inferred that the epoxy resin did not go further down even if the relaxation time was increased in nanoseconds. This is because the upper CNTs and the epoxy resin attracted each other due to interaction, and consequently, the epoxy resin did not approach the lower CNTs and graphene.

### 3.2. The Interaction Energy between One Molecule and Other Molecules

We obtained the values of the interaction energy between one molecule and other molecules. The values of $E_{G-\left(C/E\right)}$ for each model are presented in Table 2. Comparing the model with two CNTs (4,4) against the model with three CNTs (4,4), the model with three CNTs (4,4) had larger interaction energy. This indicated that the interaction energy increased as the number of CNTs increased. We inferred that more stable π–π interactions occurred between CNTs and graphene as the number of CNTs increased. Comparing the model with two CNTs (4,4) and the model with two CNTs (8,8), the model with two CNTs (8,8) had larger interaction energy. This indicated that the interaction energy increased when CNTs with a large diameter were included. This is because the bottom surface of a large-diameter CNTs was more parallel to the *xy* plane than that of a small-diameter CNTs, and thus we inferred that this was caused by more stable π–π interactions with graphene. Considering temperature, the model subjected to temperature change showed a larger value than the model subjected to a constant temperature. However, it was a numerical value that could be treated as an error. Finally, the amount of interaction energy changed depending on the arrangement of the CNTs. The interaction energy of the model with four CNTs (8,8) vertical arrangement was lower than that of the model with parallel arrangements. This is because the epoxy resin did not reach the CNTs at the bottom of the structure, and consequently, the amount of interaction with graphene was lower. The values of $E_{E-\left(C/G\right)}$ and $E_{C-\left(E/G\right)}$ are presented in Table 3 and Table 4, respectively. In this case, the larger the diameter of the CNT and the larger the number of CNTs, the larger the interaction energy. Conclusively, we considered that the CNT diameter and number of CNTs affected the interaction energy value. However, the model subjected to temperature change showed a larger value than the model subjected to a constant temperature. As before, the numerical value was a value that could be treated as an error. As above, the interaction energy value also changed depending on the arrangement of the CNTs. The result was that the interaction energy of the model with four CNTs (8,8) vertical arrangements was lower than that of the model with parallel arrangements. This is also because the epoxy resin did not reach the CNTs at the bottom of the structure.

### 3.3. The Interaction Energy between Two Individual Molecules

The interaction energy values for $E_{E-G}$, $E_{C-G}$, and $E_{E-C}$ are presented in Table 5, respectively. We compared each interaction energy value, and the result was $E_{E-C}$ > $E_{C-G}$. In other words, CNTs were more strongly attracted to epoxy resin than to graphene. It was assumed to be related to the π–π interaction between the benzenes contained in CNTs, graphene, and the epoxy resin. Concerning the interaction between CNTs and graphene, the benzene in the CNTs parallel to the *xy* plane was arranged parallel to the benzene in the graphene. The benzenes arranged in parallel show strong π–π interaction, whereas the interaction between the non-parallel benzenes shows weak π–π interaction [41]. Consequently, the parallel surfaces of CNTs-graphene were stable, and the other non-parallel inclined surfaces were unstable. As can be seen in Figure 6, the proportion of non-parallel benzenes was larger than that of parallel benzenes. Thus, we surmised that, overall, the interaction energy was in an unstable state. On the other hand, we focused on the interaction between CNTs and epoxy resin. In a previous study, Zhang Y et al. compared the interaction energies of the three benzene rings in various configurations. It was shown that a stronger π–π interaction occurred when the three benzene rings were arranged, neither parallel nor vertical to each other [42]. Consequently, the frame of the epoxy resin was flexible and could move freely in the cell; thus, the CNTs were surrounded by the epoxy resin. The benzenes in the epoxy resin could also move freely, such that there was a more stable π–π interaction with the benzene in the CNTs, and overall, it was assumed that the interaction energy was stable. Thus, we considered the π–π interaction between randomly arranged benzenes in the epoxy resin and the benzene in the CNTs to be more stable than the π–π interaction between CNTs and graphene. If the epoxy resin could flow, it could be predicted that the CNT would also flow. Finally, in the model with four CNTs (8,8), the result was $E_{E-C}$ > $E_{E-G}$. This is because it contained more CNTs than the other models, which caused a more stable π–π interaction. By comparing the values of $E_{G-\left(C/E\right)}$ and $E_{E-C}$ in the results obtained above, we found that polymer systems containing CNTs showed stronger interaction. These results indicated that the addition of CNTs had a significant effect on the interaction between the molecules.

## 4. Conclusions

We analyzed a three-phase structure composed of epoxy resin, CNTs, and graphene using MD simulation. First, we confirmed the structure of the molecules after relaxation. The epoxy resin penetrated in between the small-diameter CNTs but did not penetrate in between the large-diameter CNTs. In the model with the CNTs arranged vertically, it was observed that the epoxy resin didn’t reach the graphene because the CNTs become an obstacle. Furthermore, we obtained the interaction energy between two individual molecules. The interaction energy value increased as the CNT diameter increased and also as the number of CNTs increased. Based on $E_{E-C}$ > $E_{C-G}$, it was determined that CNTs were more strongly attracted to epoxy resin than to graphene, and it was greatly related to π–π interaction. However, we didn’t consider the condition of the electric charge in this study; we thought that the van der Waals and Coulomb forces between molecules also might have a significant effect on the interaction energy. We also surmised that the π–π interactions became stronger and exhibited higher interaction energy values as the length and number of epoxy molecular chains increased, and the distances between the molecules got closer. In this study, it was found that CNTs were attracted to the resin flow and did not adhere to the carbon fibers. In the future, we are planning to analyze the three-phase structure using epoxy resin made by cross-linking reactions.

## Figures and Tables

**Table 1:** Dimensions of simulation cell and carbon nanotube (CNT) diameter, number of CNTs, how to arrange CNTs, and temperature change values of each model.

Model	Dimensions of Simulation Cell (*x, y, z*) (Å)	CNT Diameter (nm)	Number of CNTs	Arrangements of CNTs	Temperature Changes (K)
(a) Two CNT (4,4)	(44.32, 38.38, 160.0)	0.542	2	-	-
(b) Three CNT (4,4)	(44.32, 38.38, 160.0)	0.542	3	-	-
(c) Two CNT (8,8)	(44.32, 38.38, 190.0)	1.084	2	-	-
(d) Two CNT (8,8) considering temperature changes	(44.32, 38.38, 190.0)	1.084	2	-	600→300
(e) Four CNT (8,8) parallel arrangements	(44.32, 38.38, 190.0)	1.084	4	parallel	--
(f) Four CNT (8,8) vertical arrangements	(44.32, 38.38, 190.0)	1.084	4	vertical	

**Table 2:** $E_{G-\left(C/E\right)}$ Values and the energy values used in the calculation.

	*E*_total_ (kJ/mol)	*E*_G_ (kJ/mol)	*E*_C/E_ (kJ/mol)	*E*_G−(C/E)_ (kJ/mol)
(a) Two CNT (4,4)	4.579 × 10^5^	3.401 × 10^4^	4.288 × 10^5^	4848
(b) Three CNT (4,4)	4.813 × 10^5^	3.402 × 10^4^	4.524 × 10^5^	5200
(c) Two CNT (8,8)	4.738 × 10^5^	3.402 × 10^4^	4.450 × 10^5^	5192
(d) Two CNT (8,8) considering temperature changes	4.726 × 10^5^	3.402 × 10^4^	4.440 × 10^5^	5378
(e) Four CNT (8,8) parallel arrangements	5.375 × 10^5^	3.404 × 10^4^	5.088 × 10^5^	5297
(f) Four CNT (8,8) vertical arrangements	7.261 × 10^5^	3.406 × 10^4^	6.966 × 10^5^	4504

**Table 3:** $E_{E-\left(C/G\right)}$ Values and the energy values used in the calculation.

	*E*_total_ (kJ/mol)	*E*_E_ (kJ/mol)	*E*_C/G_ (kJ/mol)	*E*_E−(C/G)_ (kJ/mol)
(a) Two CNT (4,4)	4.580 × 10^5^	3.819 × 10^5^	8.108 × 10^4^	5030
(b) Three CNT (4,4)	4.813 × 10^5^	3.823 × 10^5^	1.042 × 10^5^	5265
(c) Two CNT (8,8)	4.738 × 10^5^	3.817 × 10^5^	9.755 × 10^4^	5462
(d) Two CNT (8,8) considering temperature changes	4.726 × 10^5^	3.811 × 10^5^	9.744 × 10^4^	5917
(e) Four CNT (8,8) parallel arrangements	5.375 × 10^5^	3.823 × 10^5^	1.616 × 10^5^	6350
(f) Four CNT (8,8) vertical arrangements	7.261 × 10^5^	3.953 × 10^5^	3.363 × 10^5^	5437

**Table 4:** $E_{C-\left(E/G\right)}$ Values and the energy values used in the calculation.

	*E*_total_ (kJ/mol)	*E*_C_ (kJ/mol)	*E*_G/E_ (kJ/mol)	*E*_C−(E/G)_ (kJ/mol)
(a) Two CNT (4,4)	4.579 × 10^5^	4.823 × 10^4^	4.122 × 10^5^	2518
(b) Three CNT (4,4)	4.813 × 10^5^	7.191 × 10^4^	4.129 × 10^5^	3576
(c) Two CNT (8,8)	4.738 × 10^5^	6.522 × 10^4^	4.122 × 10^5^	3666
(d) Two CNT (8,8) considering temperature changes	4.726 × 10^5^	6.509 × 10^4^	4.114 × 10^5^	3871
(e) Four CNT (8,8) parallel arrangements	5.375 × 10^5^	1.302 × 10^5^	4.136 × 10^5^	6287
(f) Four CNT (8,8) vertical arrangements	7.261 × 10^5^	3.041 × 10^5^	4.267 × 10^5^	4632

**Table 5:** $E_{E-G}$, $E_{C-G},$ and $E_{E-C}$ Values for each of the six models.

	*E*_E−G_ (kJ/mol)	*E*_C−G_ (kJ/mol)	*E*_E−C_ (kJ/mol)
(a) Two CNT (4,4)	3681	1168	1350
(b) Three CNT (4,4)	3445	1755	1820
(c) Two CNT (8,8)	3494	1698	1968
(d) Two CNT (8,8) considering temperature changes	3712	1666	2205
(e) Four CNT (8,8) parallel arrangements	2688	2622	3662
(f) Four CNT (8,8) vertical arrangements	2655	1849	2783
